# Theory-based analysis of clinical efficacy of triptans using receptor occupancy

**DOI:** 10.1186/1129-2377-15-85

**Published:** 2014-12-08

**Authors:** Kentaro Tokuoka, Risa Takayanagi, Yuji Suzuki, Masayuki Watanabe, Yasuhisa Kitagawa, Yasuhiko Yamada

**Affiliations:** 1 Department of Neurology, Tokai University Hachioji Hospital, 1838 Ishikawa-cho, Hachioji, Tokyo 192-0032, Japan; 2 School of Pharmacy, Tokyo University of Pharmacy and Life Sciences, 1432-1 Horinouchi, Hachioji, Tokyo 192-0392, Japan; 3 Department of Pharmacy, Tokai University Hachioji Hospital, 1838 Ishikawa-cho, Hachioji, Tokyo 192-0032, Japan

**Keywords:** Triptans, Serotonin 5-HT_1B/1D_ receptor agonists, Receptor occupancy, Migraine

## Abstract

**Background:**

Triptans, serotonin 5-HT_1B/1D_ receptor agonists, exert their action by targeting serotonin 5-HT_1B/1D_ receptors, are used for treatment of migraine attack. Presently, 5 different triptans, namely sumatriptan, zolmitriptan, eletriptan, rizatriptan, and naratriptan, are marketed in Japan. In the present study, we retrospectively analyzed the relationships of clinical efficacy (headache relief) in Japanese and 5-HT_1B/1D_ receptor occupancy (Φ_1B_ and Φ_1D_). Receptor occupancies were calculated from both the pharmacokinetic and pharmacodynamic data of triptans.

**Methods:**

To evaluate the total amount of exposure to drug, we calculated the area under the plasma concentration-time curve (AUC_cp_) and the areas under the time curves for Ф_1B_ and Ф_1D_ (AUC_Ф_^1B^ and AUC_Ф_^1D^). Moreover, parameters expressing drug transfer and binding rates (*A*_*cp*_*, A*_*Ф*_^*1B*^, *A*_*Ф*_^*1D*^) were calculated.

**Results:**

Our calculations showed that Ф_max_^1B^ and Ф_max_^1D^ were relatively high at 32.0-89.4% and 68.4-96.2%, respectively, suggesting that it is likely that a high occupancy is necessary to attain the clinical effect. In addition, the relationships between therapeutic effect and AUC_cp_, AUC_Φ_^1B^, AUC_Φ_^1D^, and *A*_*cp*_ · AUC_cp_ differed with each drug and administered form, whereas a significant relationship was found between the therapeutic effect and *A*_*Φ*_^*1B*^ · AUC_Φ_^1B^ or *A*_*Φ*_^*1D*^ · AUC_Φ_^1D^ that was not affected by the drug and the form of administration.

**Conclusions:**

These results suggest that receptor occupancy can be used as a parameter for a common index to evaluate the therapeutic effect. We considered that the present findings provide useful information to support the proper use of triptans.

## Background

Triptans, serotonin 5-HT_1B/1D_ receptor agonists, exert their action by targeting serotonin 5-HT_1B/1D_ receptors, abundantly distributed in cerebral blood vessels, and are used for treatment of migraine attack [1,2]. Presently, 5 different triptans, namely sumatriptan, zolmitriptan, eletriptan, rizatriptan, and naratriptan, are marketed in Japan. Sumatriptan is available as injection, oral, and nasal preparations, while zolmitriptan, eletriptan, rizatriptan, and naratriptan are administered orally. However, differences in their therapeutic effects among individuals and drug characteristics, as well as lack of evidence for selecting a suitable drug for each patient are problems to be resolved [3]. For this reason, it is important to establish an index for quantitative evaluation of the pharmacological and clinical effects of triptans.

We have been studying the receptor occupancy, which integrates pharmacokinetic and pharmacodynamics data, of a variety of drugs that exert pharmacological effects through receptors, and theoretically evaluated clinical and adverse effects by retrospective analysis [4-7]. Our findings for sumatriptan indicate that receptor binding occupancy could be a useful parameter for clinical evaluation irrespective of the administered form [8]. In the present study, we examined whether our analytical method is applicable to all available triptans.

## Methods

We collected pharmacokinetic and pharmacodynamic parameters from administrations of sumatriptan (injectable, oral and nasal preparations), zolmitriptan (oral preparation), eletriptan (oral preparation), rizatriptan (oral preparation), and naratriptan (oral preparation) to Japanese patients as well as available clinical efficacy data. In Japan, substantial domestic clinical trial data are included in a new medicine application data package. For the present drugs identified as eligible for analysis, information regarding approved indications, characteristics of the products, and clinical trial data were extracted from review reports downloaded from the website ( http://www.info.pmda.go.jp) of the regulatory authority (Pharmaceutical and Medical Devices Agency, PMDA). When information was not provided on this website, we collected data from a relevant published article.

Receptor occupancy following each administration was calculated to investigate its relationship with clinical effect.

### Extraction of pharmacokinetic and pharmacodynamic parameters

As pharmacokinetic parameters, changes in plasma drug concentration following administration (C_p_), maximum plasma concentration (C_max_), time to maximum concentration (T_max_), plasma unbound fraction value (f_u_), and the presence of active metabolites were collected from clinical trial data obtained from Japanese subjects. For the pharmacodynamics parameters, the dissociation constant (Ki) values of the serotonin 5-HT_1B_ and 5-HT_1D_ receptors were collected.

### Extraction of clinical trial data

To determine clinical effects, changes in values for headache relief rate over time obtained in Phase II and III clinical trials were collected.

### Calculation of drug concentration and serotonin 5-HT_1B/1D_ receptor occupancy

Receptor occupancy [Ф (%)] for a particular drug was calculated using (Eq. 1) when the target drug has no active metabolite and only the unchanged drug was the active form, where C is the drug concentration (nM) near the receptor and K_i_ the dissociation constant (nM) from each receptor [9].

(1) $$\Phi \left(\%\right)=\frac{C}{C+K_{i}}\times 100$$

When only one kind of active metabolite exists, occupancy was calculated using (Eq. 2), where C_1_ and C_2_ are the drug concentrations (nM) of the unchanged drug near the receptor and active metabolite, respectively, and K_i1_ and K_i2_ are the receptor dissociation constants (nM) from each receptor of the unchanged drug and active metabolite, respectively [9].

(2) $$\begin{array}{ll} \;\Phi \left(\%\right) & =\left\{\frac{C_{1}}{C_{1}+K_{i1}\cdot \left(1+\frac{C_{2}}{K_{i2}}\right)}+\frac{C_{2}}{C_{2}+K_{i2}\cdot \left(1+\frac{C_{1}}{K_{i1}}\right)}\right\}\\ & \;\times 100 \end{array}$$

If the unbound drug in plasma is speculated to simply diffuse through the vascular wall to reach the intercellular gap and reach instant equilibrium, the drug concentration near the receptor, C, can be approximated based on the plasma unbound drug concentration (C_f_) obtained by multiplying the plasma drug concentration (C_p_) by the plasma unbound fraction (f_u_): C_f_ = C_p_ x f_u_ (nM). Furthermore, by using the 5-HT_1B_ and 5-HT_1D_ receptor dissociation constants (K_i_^1B^, K_i_^1D^), the occupancy for each subtype, Ф_1B_ and Ф_1D,_ was calculated. On the basis of data reported regarding the time course of plasma concentration after administration of each drug in Japanese subjects, the plasma unbound drug concentration (C_f_) was calculated. Then, Ф_1B_ and Ф_1D_ were calculated using Eq. 1 or Eq. 2 to examine changes over time.

### Calculation of total amount of exposure to drug and serotonin 5-HT_1B/1D_ receptor occupancy

The area under the plasma concentration-time curve (AUC_cp_) and the areas under the time curves for Ф_1B_ and Ф_1D_ (AUC_Ф_^1B^ and AUC_Ф_^1D^) from the start of drug administration to the time of evaluating headache relief [t (hr)] were calculated using the trapezoidal method.

### Calculation of systemic drug transfer and serotonin 5-HT_1B/1D_ receptor binding rates

It has been suggested that the onset of the effect of triptans is related to the systemic drug-transfer rate [10], thus parameters expressing drug transfer and binding rates (*A*_*cp*_*, A*_*Ф*_^*1B*^, *A*_*Ф*_^*1D*^) were calculated by using (Eq. 3, 4, 5), where the maximum plasma concentration was C_max_, and the time to maximum concentration T_max_ and the maximum occupancy were Ф_max_^1B^ and Ф_max_^1D^, respectively.

(3) $$A_{\text{cp}}=C_{\text{max}}/T_{\text{max}}$$

(4) $${A_{\Phi }}^{1B}={\Phi_{\text{max}}}^{1B}/T_{\text{max}}$$

(5) $${A_{\Phi }}^{1D}={\Phi_{\text{max}}}^{1D}/T_{\text{max}}$$

### Analysis of relationship between parameters for receptor occupancy and clinical effects of each drug following administration

The relationship of each parameter, obtained as noted in Section 2, with clinical effects was examined. We initially investigated the relationship of AUC_cp_, AUC_Ф_^1B^, and AUC_Ф_^1D^ with headache relief rate at each evaluation time point. Then, *A*_*cp*_ · AUC_cp_, *A*_*Ф*_^*1B*^ · AUC_Ф_^1B^, and *A*_*Ф*_^*1D*^ · AUC_Ф_^1D^, which represented the AUC values multiplied, respectively, by *A*_*cp*_, *A*_*Ф*_^*1B*^, and *A*_*Ф*_^*1D*^ obtained with the equations, Eq. 3, Eq. 4, and Eq. 5, and coupled with the rate were calculated for evaluating the relationship with headache relief rate. When an association was found between a parameter and headache relief rate, analysis was conducted by using the sigmoid Emax model shown in Eq. 6, where E is headache relief rate (%), E_max_ the maximum headache relief rate (%), X the parameters with a favorable relationship with headache relief rate, EC_50_ the value of X when E_max_ was 50%, and γ the Hill coefficient. The relationship between X and headache relief rate (E) was analyzed using a nonlinear least squares method to calculate E_max_, EC_50_, and γ. For the analyses, we used the MLAB software package (Civilized Software Inc.).

(6) $$E=E_{\text{max}}\cdot X^{\gamma }/\left({\text{EC}}_{50}+X^{\gamma }\right)$$

## Results

### Extraction of pharmacokinetic and pharmacodynamic parameters

The various parameters collected for each drug are shown in Table 1.

**Table 1:** **The various parameters for Triptans marketed in Japan**[11-20]

**Drugs**	**Administration route (dosage form)**	**Standard dose in Japan (mg)**	**MW (g/mol)**	**Plasma unbound fraction; fu**	**Ki**^1B^* (nM)	**Ki**^1D^* (nM)
Sumatriptan [11-13]	SC	3	295.41	0.66	12.6	12.6
	PO	50				
	NS	20				
Zolmitriptan [14-16]	PO	2.5	[unchanged drug]	[unchanged drug]	[unchanged drug]	[unchanged drug]
	ODT		287.30	0.8	6.31	2.51
			[active metabolite]	[active metabolite]	[active metabolite]	[active metabolite]
			273.36	0.75	1.58	0.5
Eletriptan [17]	PO	20	382.52	0.13	10.0	1.2
Rizatriptan [18,19]	PO	10	269.35	0.86	138	13.1
	ODT					
Naratriptan [20]	PO	2.5	335.47	0.71	2.2	2.3

*calculated from pKi or pIC_50._

SC: subcutaneous injection.

PO: oral tablet.

NS: nasal spray.

ODT: orally disintegrating tablet.

Ki_1B_: 5-HT_1B_ receptor dissociation constant.

Ki_1D_: 5-HT_1D_ receptor dissociation constant.

For pharmacodynamic parameters, the value of K_i_ was calculated on the basis of the reported values for pK_i_ and pIC_50_ of the 5-HT_1B_ and 5-HT_1D_ receptors. Sumatriptan and naratriptan showed nearly the same levels for those receptors, while zolmitriptan (unchanged drug and active metabolite), eletriptan, and rizatriptan tended to show a higher affinity for 5-HT_1D_ than 5-HT_1B_.

### Extraction of clinical trial data

Table 2 shows reported time courses for headache relief rate in Japanese subjects given each drug at various doses in clinical trial settings.

**Table 2:** **Time courses for headache relief (percent of patients (%)) in Japanese subjects**[11,16,21-27]

**t (Time after administration) (hr)**	**0.17**	**0.25**	**0.5**	**0.75**	**1**	**1.5**	**2**	**3**	**4**
Sumatriptan [11,21,22]									
SC 3 mg^※^	30.3	37.0-63.3	62.0-81.8	73.7	78.9-93.9	-	-	-	-
SC 6 mg	-	71.4	71.4	-	85.7	-	-	-	-
Sumatriptan [23]									
PO 50 mg^※^	-	-	7	-	32	-	41	63	68
PO 100 mg	-	-	10	-	29	-	55	65	68
Sumatriptan [24]									
NS 10 mg	-	-	19.3	-	33.3	-	58.9	-	62.5
NS 20 mg^※^	-	-	15.7-27.8	-	31.5-56.4	49.1	63.0-70.9	-	73.2
Zolmitriptan [16]									
PO 2.5 mg^※^	-	-	9.8	-	28.3	-	55.6	-	76.5
Eletriptan [25]									
PO 20 mg^※^	-	-	13	-	37	-	64	-	86
PO 40 mg	-	-	12	-	33	-	67	-	88
Rizatriptan [26]									
PO 10 mg^※^	-	-	10.1	-	26.1	50.7	59.4	-	-
Naratriptan [27]									
PO 1 mg	-	-	-	-	21.2	-	41.3	60.6	71.2
PO 2.5 mg^※^	-	-	-	-	15.6	-	50.5	71.6	77.1

※: standard dose in Japan.

SC: subcutaneous injection.

PO: oral tablet.

NS: nasal spray.

The sumatriptan injectable preparation showed a high level of headache relief from the initial stage of administration, with 60% or greater at 0.5 hours and 80% or more at 1 hour after administration. Moreover, the sumatriptan nasal preparation tended to exert its effect earlier than the other drugs. However, a relief rate of about 60-88% was attained from 2 hours with these drugs and preparations.

### Calculation of serotonin 5-HT_1B/1D_ receptor occupancy at time of administration of each drug

**Figure 1:**
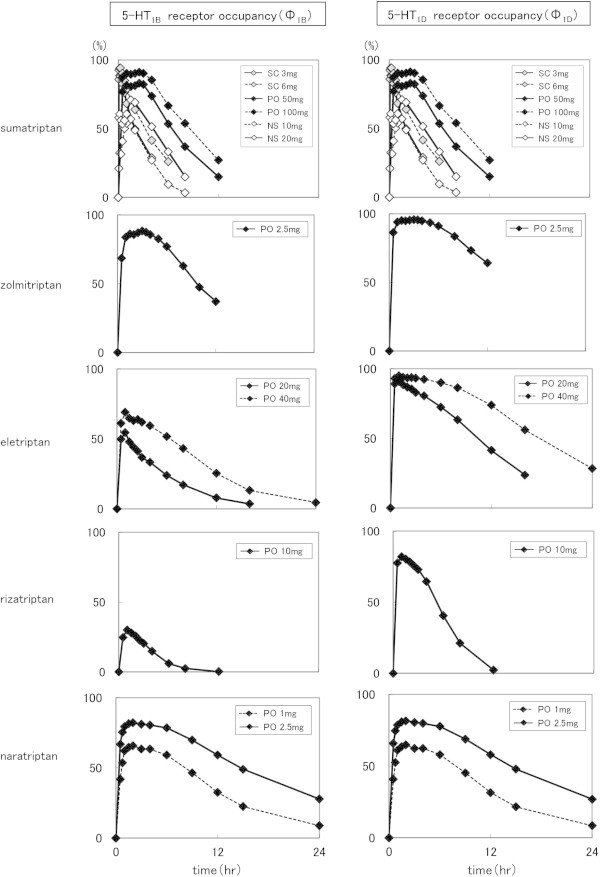
**Time courses of Ф**_**1B **_**and Ф**_**1D **_**of the tested triptans in varying doses.** (SC: subcutaneous injection, PO: oral tablet, NS: nasal spray, solid line: standard dose in Japan).

The time courses of Ф_1B_ and Ф_1D_ calculated on the basis of changes in plasma concentration after administration of the tested triptans in varying doses [11,16,23-27] demonstrated different aspects depending on the drug and dosage form (Figure 1).

Sumatriptan and naratriptan showed nearly the same transition for the 5-HT_1B_ and 5-HT_1D_ receptors, while zolmitriptan, eletriptan, and rizatriptan showed higher Ф values for the 5HT_1D_ receptor than the 5-HT_1B_ receptor.

### Calculation of total amount of exposure to drug and serotonin 5-HT_1B/1D_ receptor occupancy

Table 3 shows the AUC_cp_, AUC_Ф_^1B^, and AUC_Ф_^1D^ values from the time of administration until each evaluation time when headache relief rate was determined by the trapezoidal method.

**Table 3:** **Calculated (1) AUC**_**cp**_**, (2) AUC**_**Φ**_^**1B**^**, and (3) AUC**_**Φ**_^**1D **^**values**

**t (Time after administration) (hr)**	**0.17**	**0.25**	**0.5**	**0.75**	**1**	**1.5**	**2**	**3**	**4**
**(1) AUC**_**cp **_**(ng · hr/mL)**									
Sumatriptan									
SC 3 mg^※^	4.9	8.3	17.0	22.6	26.4	-	-	-	-
SC 6 mg	-	17.0	34.8	-	51.9	-	-	-	-
Sumatriptan									
PO 50 mg^※^	-	-	4.7	-	15.7	-	40.0	66.4	87.4
PO 100 mg	-	-	9.7	-	32.6	-	83.3	138.6	181.6
Sumatriptan									
NS 10 mg	-	-	1.2	-	4.0	-	10.7	-	18.2
NS 20 mg^※^	-	-	3.5	-	7.2	12.8	19.4	-	38.0
Zolmitriptan									
PO 2.5 mg^※^	-	-	0.51	-	1.87	-	5.4	-	11.3
Eletriptan									
PO 20 mg^※^	-	-	7.7	-	25.2	-	56.9	-	99.2
PO 40 mg	-	-	12.4	-	43.0	-	105.9	-	214.1
Rizatriptan									
PO 10 mg^※^	-	-	3.6	-	11.8	20.6	28.5	-	-
Naratriptan									
PO 1 mg	-	-	-	-	0.8	-	2.7	4.6	6.4
PO 2.5 mg^※^	-	-	-	-	2.1	-	6.6	11.2	15.6
**(2) AUC**_**Φ**_^**1B **^**(% · hr)**									
Sumatriptan									
SC 3 mg^※^	11.3	18.3	39.7	59.6	77.7	-	-	-	-
SC 6 mg	-	19.6	42.7	-	85.0	-	-	-	-
Sumatriptan									
PO 50 mg^※^	-	-	19.3	-	58.9	-	140.0	222.5	300.4
PO 100 mg	-	-	21.8	-	66.3	-	156.2	247.0	334.9
Sumatriptan									
NS 10 mg	-	-	14.5	-	38.9	-	93.2	-	169.2
NS 20 mg^※^	-	-	26.3	-	54.6	87.5	122.6	-	243.2
Zolmitriptan									
PO 2.5 mg^※^	-	-	17.1	-	55.2	-	140.7	-	314.9
Eletriptan									
PO 20 mg^※^	-	-	12.5	-	38.6	-	87.3	-	163.2
PO 40 mg	-	-	15.3	-	47.9	-	113.3	-	237.2
Rizatriptan									
PO 10 mg^※^	-	-	6.2	-	19.9	34.4	47.8	-	-
Naratriptan									
PO 1 mg	-	-	-	-	36.9	-	101.2	165.7	229.1
PO 2.5 mg^※^	-	-	-	-	53.9	-	135.1	216.7	297.6
**(3) AUC**_**Φ**_^**1D **^**(% · hr)**									
Sumatriptan									
SC 3 mg^※^	11.3	18.3	39.7	59.6	77.7	-	-	-	-
SC 6 mg	-	19.6	42.7	-	85.0	-	-	-	-
Sumatriptan									
PO 50 mg^※^	-	-	19.3	-	58.9	-	140.0	222.5	300.4
PO 100 mg	-	-	21.8	-	66.3	-	156.2	247.0	334.9
Sumatriptan									
NS 10 mg	-	-	14.5	-	38.9	-	93.2	-	169.2
NS 20 mg^※^	-	-	26.3	-	54.6	87.5	122.6	-	243.2
Zolmitriptan									
PO 2.5 mg^※^	-	-	21.6	-	66.6	-	161.2	-	351.8
Eletriptan									
PO 20 mg^※^	-	-	22.3	-	67.4	-	156.0	-	322.9
PO 40 mg	-	-	23.2	-	70.2	-	164.2	-	350.5
Rizatriptan									
PO 10 mg^※^	-	-	19.4	-	59.3	99.8	139.5	-	-
Naratriptan									
PO 1 mg	-	-	-	-	36.9	-	101.2	165.7	229.1
PO 2.5 mg^※^	-	-	-	-	53.9	-	135.1	216.7	297.6

※: standard dose in Japan.

SC: subcutaneous injection.

PO: oral tablet.

NS: nasal spray.

### Calculation of systemic drug-transfer rate and rate of serotonin 5-HT_1B/1D_ receptor binding

Table 4 shows calculated values for T_max_, C_max_, the absorption rate-related parameters *A*_*cp*_ (C_max_/T_max_), Ф_max_^1B^, Ф_max_^1D^, and the receptor occupancy rate-related parameters *A*_*Ф*_^*1B*^ (Ф_max_^1B^/T_max_) and *A*_*Ф*_^*1D*^ (Ф_max_^1D^/T_max_).

**Table 4:** **Pharmacokinetic parameters and calculated values**[11,16,23-27]

	**T**_**max **_**(hr)**	**C**_**max **_**(ng/mL)**	***A***_cp_* (C_**max**_**/T**_**max**_**)**	**Φ**_**max**_^1B ^* (%)	**Φ**_**max**_^1D ^* (%)	***A***_***Φ***_^1B ^* (Φ_**max1B**_**/T**_**max**_**)**	***A***_***Φ***_^1D ^* (Φ_**max1D**_**/T**_**max**_**)**
Sumatriptan [11]							
SC 3 mg^※^	0.21	44	209.52	88.6	88.6	422.09	422.09
SC 6 mg	0.2	95.5	477.50	94.4	94.4	472.12	472.12
Sumatriptan [23]							
PO 50 mg^※^	1.8	32.6	18.11	85.3	85.3	47.36	47.36
PO 100 mg	2	58.2	29.10	91.2	91.2	45.58	45.58
Sumatriptan [24]							
NS 10 mg	1.1	6.4	5.82	53.2	53.2	48.33	48.33
NS 20 mg^※^	1.3	12.2	9.38	68.4	68.4	52.61	52.61
Zolmitriptan [16]							
PO 2.5 mg^※^	[unchanged drug]	[unchanged drug]	[unchanged drug]	89.4	96.2	26.79	32.1
	3	5.23	1.74				
	[active metabolite]	[active metabolite]	[active metabolite]				
	3	3.5	1.17				
Eletriptan [25]							
PO 20 mg^※^	1	38.9	38.90	56.9	91.7	56.93	91.7
PO 40 mg	1.2	69.7	58.08	70.3	95.2	58.6	79.3
Rizatriptan [26]							
PO 10 mg^※^	1	20.3	20.30	32.0	83.2	32.0	83.2
Naratriptan [27]							
PO 1 mg	2.17	2.12	0.98	67.1	66.1	30.9	30.5
PO 2.5 mg^※^	2.68	5.62	2.10	84.4	83.8	31.5	31.3

*: calculated, ※: standard dose in Japan.

SC: subcutaneous injection.

PO: oral tablet.

NS: nasal spray.

T_max_: time to C_max_.

C_max_: peak plasma concentration.

Φ_max_^1B^: peak Φ_1B_.

Φ_max_^1D^: peak Φ_1D_.

For T_max_, the sumatriptan injectable preparation was the fastest at about 0.2 hours, followed by the nasal preparation at about 1 hour and the tablet at about 1-3 hours. As for Ф, Ф_max_^1B^ at the standard dose of each drug ranged from 32.0-89.4%, while Ф_max_^1D^ ranged from 68.4-96.2%.

### Analyses of relationships between parameters for receptor occupancy and clinical effects at the time of administration of each drug

**Figure 2:**
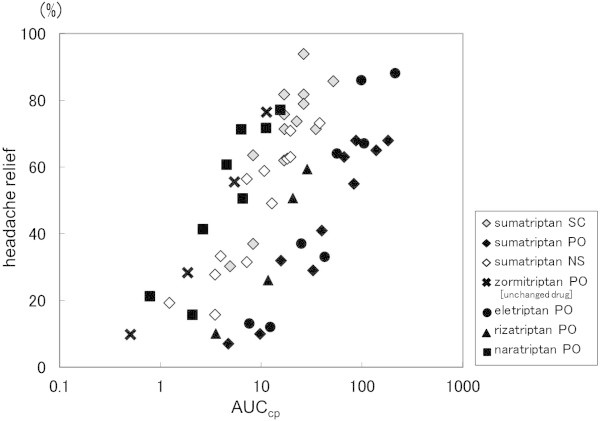
**Relationship between AUC**_**cp **_**and headache relief rate.** (SC: subcutaneous injection, PO: oral tablet, NS: nasal spray).

We examined the relationships between the parameters obtained as noted in Section 2 above and clinical effects. As for that between AUC_cp_ and headache relief rate, relief rate increased along with AUC_cp_ increases. However, that relationship tended to vary among the tested drugs (Figure 2).

**Figure 3:**
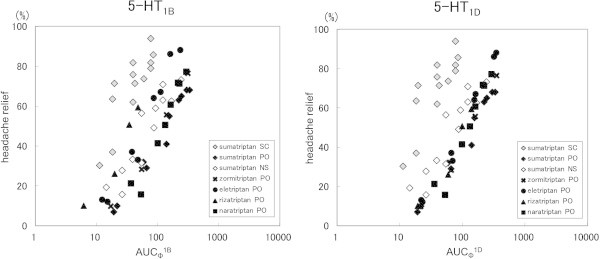
**Relationships between AUC**_**Φ**_^**1B **^**or AUC**_**Φ**_^**1D **^**and headache relief rate.** (SC: subcutaneous injection, PO: oral tablet, NS: nasal spray).

For the relationship of AUC_Ф_^1B^ and AUC_Ф_^1D^ with headache relief rate, the elevation of both was related to increased headache relief rate, with different tendencies depending on the drug. We also found that a relationship with 5-HT_1D_ receptors was likely for the oral preparations irrespective of drug, while the injectable and nasal preparations showed different aspects (Figure 3).

**Figure 4:**
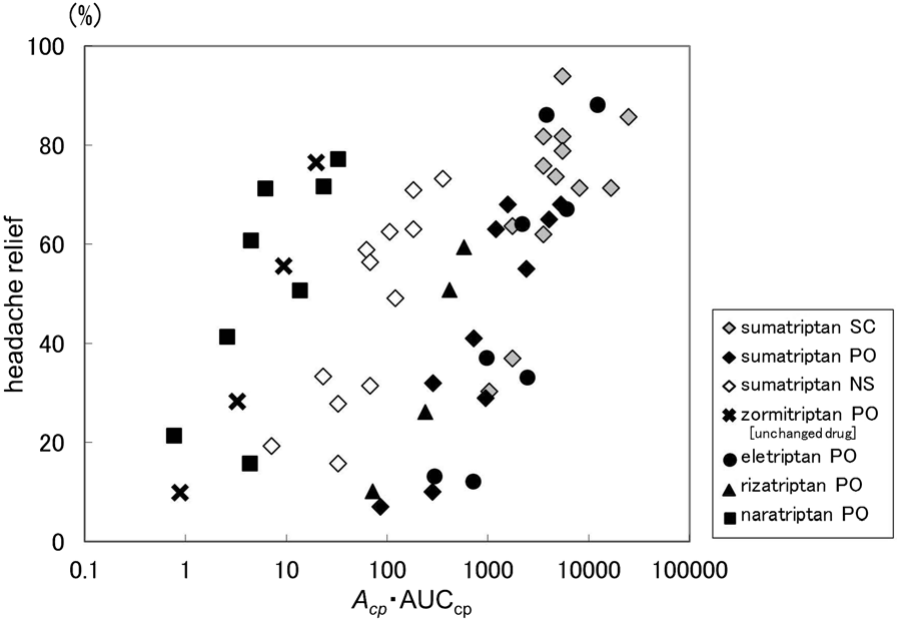
**Relationship between *A***_***CP***_ **·** **AUC**_**CP **_**and headache relief rate.** (SC: subcutaneous injection, PO: oral tablet, NS: nasal spray).

Figure 4 shows the relationship between AUC_cp_ (*A*_*cp*_ · AUC_cp_), in which the value for plasma drug concentration-related *A*_*cp*_ was coupled with velocity, and headache relief rate.

In the analysis using *A*_*cp*_ · AUC_cp_, headache relief rate was increased along with the increase in A_*cp*_ · AUC_cp_. However, the relationship varied depending on the drug. Meanwhile, the relationship between AUC_Ф_ (*A*_*Ф*_^*1B*^ · AUC_Ф_^1B^ and *A*_*Ф*_^*1D*^ · AUC_Ф_^1D^), in which receptor occupancy-related *A*_*Ф*_ was coupled with velocity, was likely to be assimilated into a single line.

**Figure 5:**
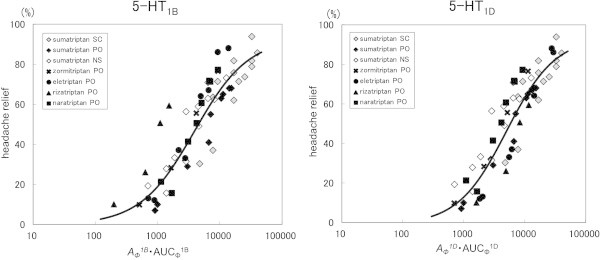
**Relationships between *A***_***Φ***_^***1B***^ **·** **AUC**_**Φ**_^**1B **^**or *A***_***Φ***_^***1D***^ **·** **AUC**_**Φ**_^**1D **^**and headache relief rate.** (SC: subcutaneous injection, PO: oral tablet, NS: nasal spray, Solid line: Fitting curve).

As a result of our analysis using the sigmoid E_max_ model, the fitted curve of our model corresponded well to the actual measurement value (Figure 5). The correlation coefficient was 0.90 for the 5-HT_1B_ receptor and 0.92 for the 5-HT_1D_ receptor. The parameters obtained are shown in Table 5.

**Table 5:** **Estimated value of E**_**max**_**, EC**_**50**_**, and γ**

**Parameter**	**Estimated value ± S.E.**	
	***A***_***Φ***_^***1B***^ **·** **AUC**_**Φ**_^**1B**^	***A***_***Φ***_^***1D***^ **·** **AUC**_**Φ**_^**1D**^
E_max_	92.5 ± 9.9	92.5 ± 8.5
EC_50_	3529.7 ± 985.6	4740.0 ± 995.7
γ	0.97 ± 0.18	1.11 ± 0.18

Both *A*_*Ф*_^*1B*^ · AUC_Ф_^1B^ and *A*_*Ф*_^*1D*^ · AUC_Ф_^1D^ could be assimilated into a single line irrespective of drug kind and dosage form, though the correlation was higher for the 5-HT_1D_ receptor than the 5-HT_1B_ receptor.

## Discussion

In our previous study, as an attempt to establish information for the optimal use of triptans, we focused on sumatriptan, which has multiple drug formulations available [8]. Our results clarified that the parameter *A*_*Ф*_ · AUC_Ф_, in which the receptor binding occupancy of the action target, the 5-HT_1B/1D_ receptor, and occupancy rate were taken into account, was a good index for quantitatively evaluating clinical efficacy without regard to dosage form. In the present study, for the purpose of evaluating whether our method is applicable for analysis of triptans in general, we examined values obtained when various triptan preparations were administered to Japanese subjects.

In principle, the pharmacokinetic data were collected from clinical trial data obtained from Japanese subjects. For zolmitriptan, the presence of an active metabolite, which contributes to drug efficacy, was taken into account in the analysis. Moreover, for eletriptan and rizatriptan, the presence of an active metabolite was not taken into account, as its contribution to efficacy is reported to be low.

For the pharmacodynamic data, the value of K_i_, which represents affinity for the 5-HT_1B_ and 5-HT_1D_ receptors, was investigated. Generally, it is desirable to use data measured simultaneously in identical experimental systems. However, since no such data were available, we used values previously reported for each drug. More precise analysis might be achieved in the future if K_i_ values measured under the same condition could be obtained. The K_i_ values varied among the drugs. Also, the degree of affinity for the 5-HT_1B_ and 5-HT_1D_ receptors was found to vary depending on the drug.

For the present analysis, the drug concentration near the target receptors was considered to be at the same level as that of the unbound drug in plasma. It was previously reported that the 5-HT_1B_ receptor, an action target of triptans, is present in the intra-cranial vessels, while the 5-HT_1D_ receptor exists in the trigeminal nerve around the intra-cranial vessels [28]. However, there are no known reports of direct measurements of drug concentration near these receptors. Accordingly, we speculated that a drug unbound from plasma protein may reach the action site by permeation through the vessel wall after being transferred to blood following administration.

The blood-brain barrier (BBB) penetration of triptan differs in each drug. Sumatriptan and naratriptan especially are scarcely passing through BBB and showing a thin distribution in the central nervous system [20,29]. However, they elicited the same therapeutic effect for migraine as other triptans. Furthermore, Tomita et al. suggested that triptans also acted on 5-HT_1B/1D_ receptors in the trigeminal and dorsal root ganglion cells where BBB was lacked rather than in the sites with BBB in the trigeminovascular system [30]. For this reasons, in this study, we have taken into account only vascular permeability of triptans. In future, the target tissue and receptor of triptans become clearer. And if actual measurement drug concentration at the target site is obtained, we will be able to do more accurate analysis in consideration of BBB penetration.

Using the collected data, changes in receptor occupancy, shown by Ф_1B_ and Ф_1D_, were calculated on the basis of reported changes in plasma drug concentration. As shown in Figure 1, sumatriptan and naratriptan had nearly the same changes for both the 5-HT_1B_ and 5-HT_1D_ receptors, as our results revealed nearly the same K_i_ values for those receptors. Meanwhile, zolmitritan, eletriptan, and rizatriptan demonstrated changes when Ф was higher for the 5-HT_1D_ receptor than for the 5-HT_1B_ receptor, as the affinity for the 5-HT_1D_ receptor was greater.

Using reported C_max_ values, we calculated Ф_max_^1B^ and Ф_max_^1D^. As shown in Table 4, the C_max_ values varied greatly depending on the drug. Meanwhile, as a certain effect was obtained without regard to the kind and dosage form, it was considered difficult to quantitatively predict the clinical effect on the basis of the plasma drug concentration. Our calculations showed that Ф_max_^1B^ and Ф_max_^1D^ were relatively high at 32.0-89.4% and 68.4-96.2%, respectively, suggesting that it is likely that a high occupancy is necessary to attain the clinical effect. Sumatriptan showed a greater than 3.6-fold difference for C_max_ between the injectable (3 mg) and nasal (20 mg) preparations at the usual dose. However, the difference between Ф_max_^1B^ and Ф_max_^1D^ was about 1.3-fold, which was not as great as that seen in regard to blood concentration. On the basis of these findings, we considered that Ф could be quantitatively indicative of the clinical effect, which was difficult to evaluate by using plasma drug concentration. Furthermore, the value for Ф necessary for the onset of efficacy could be attained in all of the dosage forms. Thus, it is suggested that Ф might be useful for quantitative evaluation of the clinical efficacy of each dosage form.

We have reported many papers about the relationships between the receptor binding occupancies and clinical effects or adverse reactions in the various kinds of drugs. Among these articles, for benzodiazepines, dopamine D_2_ antagonists, dopamine D_2_ agonists, and histamine H_1_ antagonists, we reported that theoretical calculated values reflected the actual values measured by Positron Emission Tomography (PET) [10,31-34]. Also, with regard to other drugs, we suggested that it was possible to analyze the effects of drugs by using theoretical calculated values [4-7,35].

We also examined the relationship between clinical effect and Ф. Data for the time course of clinical effect were mainly extracted from the results of Phase II and III clinical trials performed in Japan. Meanwhile, in pharmacokinetic data mainly obtained in Phase I trials of healthy adults, bioequivalence was shown to be obtained between healthy adults and patients with migraine when a tablet preparation was used [36]. Accordingly, in the present study we used data for our analyses based on the speculation that they were the same as those obtained when tablets were given to patients. In clinical trials of migraine therapeutic agents, headache relief rate at 2 hours after administration is typically used as a major evaluation time point. In the present study, with the time factor taken into account, change in headache relief rate was used as a secondary end point.

In our analysis of the relationship with headache relief rate, a different profile was suggested between drug kind or dosage form for AUC_cp_, AUC_Ф_^1B^, and AUC_Ф_^1D^, which showed no definite relationship. For the parameter *A*_*cp*_ · AUC_cp_, in which velocity was taken into consideration, differences were observed among the preparations. However, for the parameters *A*_*Ф*_^*1B*^ · AUC_Ф_^1B^ and *A*_*Ф*_^*1D*^ · AUC_Ф_^1D^, for receptor occupancy coupled with velocity, a relationship was observed irrespective of drug kind and dosage form, which could be assimilated into a single line. Moreover, the 5-HT_1D_ receptor showed a better correlation coefficient than the 5-HT_1B_ receptor. It was previously reported that the contribution to clinical efficacy varied between the 5-HT_1B_ and 5-HT_1D_ receptors [37]. Our findings suggest that the 5-HT_1D_ receptor has a greater contribution, though additional study is necessary.

Our findings indicate that is difficult to evaluate the relationship of triptan preparations with clinical efficacy by using only plasma drug concentration. However, that could be evaluated by using receptor occupancy. Furthermore, clinical efficacy might be determined with higher precision by taking into account the rate of receptor binding. Thus, we considered that the parameter *A*_*Ф*_^1D^ · AUC_Ф_^1D^ is a useful index for evaluating clinical effect.

It is likely that the clinical efficacy of triptans can not be accurately evaluated by using plasma drug concentration, if a drug such as zolmitriptan with an active metabolite is analyzed only based on the unchanged drug concentration. In light of this, evaluation on the basis of receptor occupancy, which can quantify by taking into consideration the presence of the active metabolite, is considered appropriate.

A previous report [10] suggested that absorption rate should be taken into consideration when examining the clinical efficacy of sumatriptan and the present results support those findings. Moreover, to set a parameter that takes into consideration absorption rate, a prior study [38] investigated that of sumatriptan at the time of initial administration by using C_max_/T_max_. The values reported in that study were similar to our results. Accordingly, it is considered that our method for taking into consideration velocity might be appropriate.

## Conclusions

We suggest a common index that enables evaluation of the clinical efficacy of triptans irrespective of drug kind and dosage form by using a single line. We concluded that the preparation choice and prediction of clinical effect of an additional dose for appropriately designing an administration plan can be realized by use of a standard curve. Thus, by calculating *A*_*Ф*_ and AUC_Ф_ for attaining the full clinical effect, changes in plasma drug concentration can be obtained, making possible an estimation based on drug, dosage form, and dose. Receptor occupancy can be used as a parameter for a common index to evaluate the therapeutic effect. We considered that the present findings provide useful information to support the proper use of triptans. In a future study, we intend to develop a system for clinical application.

## Abbreviations

5-HT: 5-hydroxytriptamine; AUC: Area under the curve; Φ: Receptor occupancy.

## Competing interests

The authors declare that they have no competing interests.

## Authors’ contributions

KT conceptualized and designed the study, acquired and analysis the data, and drafted the manuscript. RT conceptualized and designed the study, acquired and analysis the data, and drafted the manuscript. YS conceptualized and designed the study. MW conceptualized and designed the study. YK critically revised the manuscript. YY conceptualized and designed the study and critically revised the manuscript. All authors read and approved the final manuscript.

## Funding

No grants or fellowships are supporting the writing of the paper.
